# Reframing Formalin: A Molecular Opportunity Enabling Historical Epigenomics and Retrospective Gene Expression Studies

**DOI:** 10.1111/1755-0998.14065

**Published:** 2025-01-02

**Authors:** Clare E. Holleley, Erin E. Hahn

**Affiliations:** ^1^ National Research Collections Australia Commonwealth Scientific Industrial Research Organisation Canberra Australian Capital Territory Australia

**Keywords:** chromatin accessibility, DNA, epigenetics, formaldehyde, formalin‐fixed, gene expression, genome, museomics, museum, museum epigenomics

## Abstract

Formalin preservation of museum specimens has long been considered a barrier to molecular research due to extensive crosslinking and chemical modification. However, recent optimisation of hot alkaline lysis and proteinase K digestion DNA extraction methods have enabled a growing number of studies to overcome these challenges and conduct genome‐wide re‐sequencing and targeted locus‐specific sequencing. The newest, and perhaps most unexpected utility of formalin preservation in archival samples is its ability to preserve in situ DNA‐protein interactions at a molecular level. Retrieving this signal provides information about the relative compaction or accessibility of the genome to the transcriptional machinery required for gene expression. Thus, exposure to formalin essentially corresponds to taking a snapshot of organism‐wide gene expression at the time of death. While DNA methylation and RNA‐Seq analyses of dried tissues have provided glimpses into historical gene regulation, these techniques were previously limited to skeletal or desiccated remains, offering only partial insights. By examining fluid‐preserved specimens, molecular tools can now be applied to a broader range of tissues, enabling more detailed tissue‐specific gene regulation profiling across vertebrates. In this review, we chronicle the historical use of formaldehyde in collections and discuss how targeted chromatin profiling with assays like MNase‐seq and FAIRE‐seq are surmounting fixation challenges and unlocking invaluable insights into historical genomes and gene expression profiles. The deeper integration of molecular genetics with museum collections bridges the gap between past and present and provides a vital tool that could help us predict and mitigate some of the impacts of future environmental change, novel pathogens, or invasive species.

## 1 Introduction

Formaldehyde in its various forms, including formalin (a 3.7% aqueous solution of formaldehyde), has long been used as a chemical tool by naturalists and biologists to fix and preserve tissues and cells (Kiernan 2000). Formaldehyde use has enabled detailed study of biological structures on the scale of whole organisms down to cellular processes at single‐nucleotide resolution (Rhee and Pugh 2011; Simmons 2014). In the field of chromatin biology, formaldehyde is routinely used to induce cross‐links between DNA and proteins to study their interaction (Hoffman et al. 2015). In natural history collections, however, a dogma has emerged that formalin preservation renders biological specimens unsuitable for molecular work (Wandeler, Hoeck, and Keller 2007; Thomas 1994). Thus, until recently, formalin‐preserved museum specimens have typically been viewed as devoid of sequenceable DNA.

**FIGURE 1:**
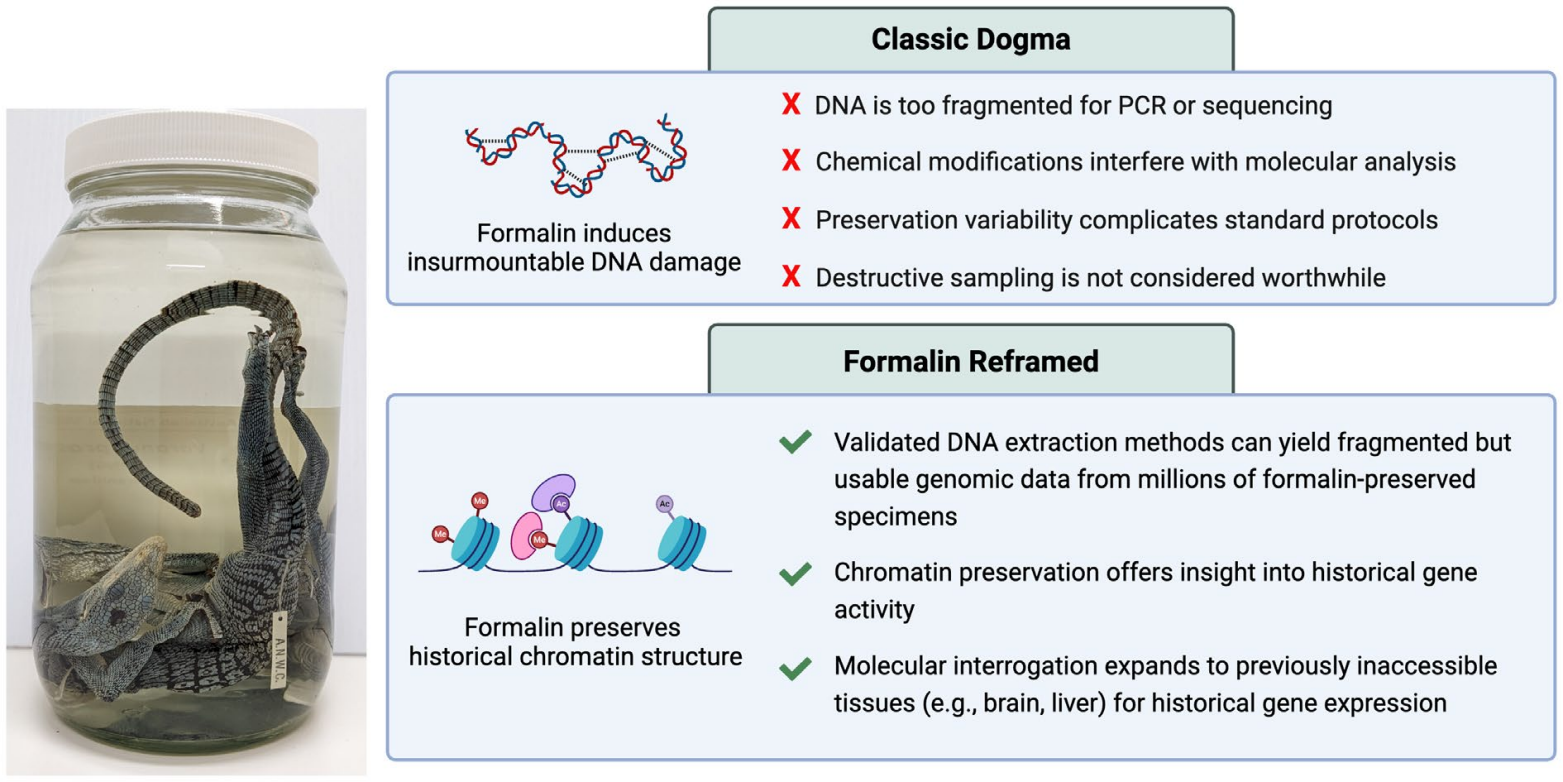
Re‐framing formalin from DNA damage to genomic opportunity. Contrasted with the classic dogma that formalin preservation inhibits molecular interrogation, our reframed perspective highlights the potential held within formalin‐preserved museum specimens such as these emerald tree monitors ( *Varanus prasinus* ) collected in Papua New Guinea in 1969 (ANWC Reg no. R01048) and 1971 (ANWC Reg no. R01065). Millions of formalin‐preserved specimens, long viewed as intractable for molecular work, can now be viewed as valuable repositories of molecular data. In addition to yielding DNA sequence data, formalin‐fixed specimens preserve genome‐wide chromatin architecture. These advancements elevate the molecular potential of formalin‐fixed specimens, and their utility may one day exceed the diversity of applications currently observed for dried and ethanol‐preserved collections. Formalin‐fixed specimens are uniquely poised to enable characterisation of historical gene activity across a broad range of tissues.

A recent study (Hahn, Stiller, et al. 2024) made the conceptual advance of linking the fields of chromatin biology and museum genomics through the common application of formalin‐fixation. Coupled with an innovative molecular approach to overcome the challenges of working with very old specimens, a tractable and reproducible method of estimating historical gene expression (via chromatin accessibility) has emerged. Historical museum practices of formalin‐fixation can now be viewed as a large‐scale untapped molecular opportunity rather than an impediment (Figure 1).

Historically, the preservation of biological materials in liquids (e.g. vinegar, brine, oil, and ethanol) dates back thousands of years, with field specimen preservation in ethanol becoming common in the 1600s (Simmons 2014). Early naturalists used liquid preservation to store voucher specimens, enabling detailed morphological descriptions and documenting the biodiversity of local and explored regions. In the early 1900s, formalin was introduced as an effective method to rapidly preserve soft tissue structure for downstream analysis in fields such as anatomy and histopathology (Simmons 2014). As a result, many of the earliest collected vertebrate specimens (including taxonomic ‘type’ specimens), were preserved in liquids. Many of these specimens were either initially fixed with formalin or later came into contact with media containing formaldehyde. Liquid preservation, particularly with formalin, is most common in taxa for which dry preservation produces subpar morphological specimens (e.g., fish, amphibians, and reptiles), but is also applied regularly to mammals and birds. A key advantage of fluid preservation is that it allows for specimens to be preserved whole, thus facilitating the study of internal organs.

Over the course of 50 years, advances in molecular genetics have transformed our ability to extract and analyse DNA from historical museum specimens. Initially limited to single‐gene Sanger DNA sequencing (Sanger, Nicklen, and Coulson 1977), researchers can now routinely reconstruct entire genomes using high‐throughput sequencing technologies that generate gigabases of data (Heather and Chain 2016; Yeates, Zwick, and Mikheyev 2016; Benham and Bowie 2023; Card et al. 2021). These advancements have fuelled progress in conservation and evolutionary science, allowing more detailed phylogenetic studies, population genetics, and the examination of genetic diversity across both time and space (Wandeler, Hoeck, and Keller 2007; Bi et al. 2013; Holmes et al. 2016; Bieker and Martin 2018; Lopez et al. 2020; Billerman and Walsh 2019). Museum specimens, once considered untapped resources, are now recognised as invaluable resources for studying species evolution, population bottlenecks, and the impacts of environmental changes on biodiversity (Raxworthy and Smith 2021; Nakahama 2021). In temporal genomics studies, these specimens enable direct tracking of genetic changes over time, revealing insights into recent adaptive responses, shifting population dynamics and the evolutionary consequences of climate change and human activity (Clark et al. 2023; Kim et al. 2023).

Meanwhile, the field of chromatin biology has advanced through the advent of techniques such as Chromatin Immunoprecipitation (ChIP) (Robertson et al. 2007), DNase I hypersensitive site sequencing (DNase‐seq) (Boyle et al. 2008), Formaldehyde Assisted Isolation of Regulatory Elements (FAIRE) (Giresi et al. 2007) and Assay for Transposable Accessible Chromatin sequencing (ATAC‐seq) (Buenrostro et al. 2013). The chromatin‐contact genome assembly approach Hi‐C (Lieberman‐Aiden et al. 2009; Dudchenko et al. 2017) reduces the cost of high‐quality telomere‐to‐telomere genome assemblies (particularly for non‐model organisms) while micrococcal nuclease sequencing (MNase‐seq) (Cui and Zhao 2012; Schones et al. 2008) enables nucleotide‐resolution characterisation of 3D‐genome structure and topological associated domains. Collectively, and through coordinated international consortiums such as the Encyclopedia of DNA Elements (ENCODE), these advances have revolutionised our understanding of genome evolution, gene function, and the plastic interaction of genes and the environment (ENCODE Project Consortium 2012; ENCODE Project Consortium et al. 2020; Hoencamp et al. 2021).

Despite advancements in sequencing historical DNA and using formalin fixation to study fine‐scale chromatin structure, formalin‐preserved museum specimens have largely been left out of genomic research. Integrating these specimens into current molecular frameworks could enhance our understanding of historical biological processes and provide insights into how species have responded to environmental changes over time (Ruane 2021).

## 2 Formalin Solutions

Only recently has the molecular potential of formalin‐preserved specimens been revisited with the help of specialised molecular and bioinformatic approaches (Hahn et al. 2022; Hahn, Alexander, et al. 2024; O'Connell et al. 2022; Ruane and Austin 2017; Hykin, Bi, and McGuire 2015; Straube et al. 2021). Standard DNA extraction methods, including those developed for formalin‐fixed paraffin‐embedded (FFPE) tissues (Robbe et al. 2018; Stiller et al. 2016) are often inappropriate for museum specimens (Shedlock et al. 1997). This is partly because museum specimens are exposed to formalin for extended periods (ranging from days to decades), unlike the more standardised fixation of FFPE samples, which are typically fixed for a specific duration, such as 48 h (Simmons 2014; Canene‐Adams 2013). Moreover, many museum specimens are not thoroughly washed after fixation and older specimens were sometimes fixed using unbuffered formalin. While these practices efficiently preserve tissues for morphological study, they lead to heavier cross‐linking, higher residual formaldehyde content, and acidification of the media over time which results in degradation of the nucleic acids (Simmons 2014; Miething et al. 2006).

Accessing and sequencing the heavily crosslinked and degraded DNA within formalin‐preserved museum specimens requires a specialised approach. Vetting tissues for sequencing suitability is essential to avoid fruitless destructive sampling of irreplaceable specimens. Specimen metadata rarely includes details describing the fixation process (e.g. time interval between death and fixation, fixative concentration, buffering agents, fixation duration, and washing protocol), and un‐fixed specimens are sometimes stored in the same jar with fixed specimens, resulting in crosslinking via formaldehyde leached into the media. Therefore, *post hoc* evaluation of tissue integrity, residual formaldehyde content, and media pH is crucial for determining specimen preservation state (Hahn et al. 2022; Hahn, Alexander, et al. 2024). Suitable specimens for molecular analysis are typically those that show high tissue integrity, indicating minimal postmortem decay, and have been stored in neutral media (with a pH between 6 and 8) (Hahn et al. 2022). With high‐quality formalin‐preserved specimens, specialised extraction methods such as hot alkaline lysis (Hahn et al. 2022; Hahn, Alexander, et al. 2024; Hykin, Bi, and McGuire 2015) or modified proteinase K digestion (O'Connell et al. 2022; Ruane and Austin 2017) have successfully recovered sequenceable DNA.

Studies that utilise short molecular barcodes have recovered mitochondrial sequences from formalin‐preserved specimens, enabling genetic identification of individuals from larval fish assemblages (Appleyard et al. 2022) and COI barcoding of fish (Zhang 2010) and invertebrate specimens (Baird et al. 2011; Jaksch et al. 2016). This approach has been extended to nuclear loci, with nuclear barcodes recovered from polychaete worms (Palmer 2008) and plankton (Shiozaki et al. 2021), and microsatellites amplified from leopard skin (Bibi et al. 2015), snow leopard tissue (Joshi et al. 2013), and box turtle claw clippings (Lutterschmidt, Cureton II, and Gaillard 2010). To broaden genomic cover, target capture sequencing has been used to construct phylogenies for snake (Ruane and Austin 2017; Bernstein and Ruane 2022) and fish species (Agne et al. 2022; Scarsbrook et al. 2023), taxa that are almost exclusively formaldehyde‐exposed. An added benefit of target capture is the recovery of off‐target mitochondrial reads as ‘bycatch’, providing additional data for phylogenetic analysis (Ruane and Austin 2017; Bernstein et al. 2023; Deepak, Ruane, and Gower 2018). Shotgun sequencing of highly fragmented DNA from formalin‐preserved specimens has also been used to recover historical whole mitochondrial haplotypes (Hahn et al. 2022; Muschick et al. 2022) and low‐coverage nuclear genomes (Hahn et al. 2022). Similar to ancient DNA (Xu et al. 2021; Jónsson et al. 2013; Kircher 2012), sequence reads from formalin‐preserved tissues are invariably degraded with lower endogenous content, necessitating tailored bioinformatic approaches such as trimming‐free alignment which has achieved mapping rates up to 58% (Hahn, Stiller, et al. 2024; Hahn et al. 2022).

Recognising the potential for nucleic acid preservation in formalin‐fixed specimens, several groups have extended their reach to surveying preserved RNA. Extracts from preserved bat specimens yielded RNA mapping to the species' reference transcriptome (Speer et al. 2022), and viral RNA has been recovered from preserved mosquitos (Lumley et al. 2021). Together, these early indicators suggest formalin‐preserved museum specimens may be invaluable resources for reconstructing historical gene expression profiles as well as host‐pathogen interactions. This promise is further supported by results with historical FFPE specimens such as successful viral and host RNA sequencing from 1918 influenza patient samples (Patrono et al. 2022; Xiao et al. 2013).

## 3 Re‐Framing Formalin

Formalin fixation presents challenges for molecular research that can be overcome with appropriate specimen vetting and application of specialised extraction and bioinformatic techniques (Hahn et al. 2022; Hahn, Alexander, et al. 2024). Collectively, the curatorial and genomics communities are beginning to re‐think the utility of formalin‐preserved specimens in yielding historical genomic data. Our group expanded on this by developing the first method to survey historical gene expression via chromatin architecture profiling (Hahn, Stiller, et al. 2024). Rather than viewing formalin as an obstacle, we took an approach of working with the opportunities presented by formalin preservation. Recognising formalin's mode of action to induce cross‐links and capture DNA‐protein interactions, we adapted established chromatin biology methods to provide unique insight into chromatin structure and thus gene expression in formalin‐preserved specimens.

While both formalin‐preserved museum specimens and tissues for chromatin assays such as ChIP‐Seq rely on formalin for fixation, there are significant differences in their preparation. Museum specimens are typically exposed to 10% formalin for extended periods, ranging from days to decades, whereas tissues and cell culture samples used for chromatin assays are fixed with lower formalin concentrations (1%–2%) for much shorter durations (minutes to hours), which minimises DNA degradation while preserving protein‐DNA interactions (Cotney and Noonan 2015). To retrieve historical chromatin profiles from archival specimens, we applied our specimen vetting system (Hahn et al. 2022) and incorporated tissue homogenisation and de‐crosslinking optimisations into existing MNase‐Seq and FAIRE‐Seq protocols to contend with heavier fixation. With the resulting sequence data, we developed an analytical framework that exploits heavy fixation to measure relative chromatin accessibility and infer gene activity.

We demonstrated the successful extraction of chromatin profiles from formalin‐preserved C57BL/6 laboratory strain mice ( *Mus musculus* ) stored for 4.8 years, wild‐caught mice stored for 2.3 years, and Australian eastern water dragons ( *Intellagama lesueurii lesueurii* ) collected between 1905 and 2001 (up to 117 years old). We showed that the archival gene expression profiles are predictive of biological state, including tissue of origin and sex, and could distinguish population background by differentiating laboratory mice from wild‐caught mice, and eastern water dragons collected from urban versus rural locations. Notably, the optimised MNase‐Seq assay simultaneously delivered archival chromatin conformation data, detected as differential occupancy values calculated from mapped sequencing depth, alongside whole genome sequencing data. Mean genomic coverage among the six mouse specimens ranged from 11.9 to 15.3×, while among the five eastern water dragons it ranged from 3.2 to 8.1×. These results underscore the potential of our approach to unlock previously inaccessible historical gene expression data from heavily formalin‐fixed specimens, opening new avenues for ‘multi‐omics’ genomic and epigenomic studies in natural history collections while minimising destructive sampling (Hahn, Stiller, et al. 2024).

Ancient epigenetic studies that estimate DNA methylation or nucleosome occupancy are generally restricted to skeletal remains and methods cannot be easily applied to other tissue‐specific signals (Orlando and Willerslev 2014; Pedersen et al. 2014; Orlando, Gilbert, and Willerslev 2015; Rubi, Knowles, and Dantzer 2020). However, stringent data filtering and cross‐species comparative genome datasets can surmount this challenge to reveal ancient regulatory evolution. For example, a study of modern humans, Neanderthals, Denisovans and chimpanzees identified methylation signatures unique to modern humans in genes responsible for vocal and facial anatomy (Gokhman et al. 2020). There are also a few examples of retrieving functional genomic data from high‐value extinct species, although these rely on rare and opportunistic preservation conditions (e.g. permafrost‐preserved mammoths (Sandoval‐Velasco et al. 2024)) or costly deep sequencing of very degraded specimens that are unlikely to yield quantitative data (e.g. transcriptome and miRNA characterisation from dried extinct Thylacine skin (Marmol‐Sanchez et al. 2023)). In comparison, our methodology is the first that offers an opportunity to estimate historical gene expression across the millions of formalin‐preserved specimens held in collections, spanning a broader range of tissues, and assessing timespans of more than a century. With further development, protocols such as our historical chromatin profiling approach will transform wet biological collections into a comprehensive and global record of environmental impacts on gene expression and phenotype (Hahn, Stiller, et al. 2024).

The increasing accessibility of molecular data from formalin‐preserved museum specimens provides novel opportunities to explore the intricacies of evolutionary processes and biodiversity through time, providing insight into the rate and frequency of change (Snead and Clark 2022). These specimens capture not only the genetic blueprint of an organism but also its gene regulatory landscape at the time of death. By enabling access to gene expression profiles, archival epigenomics offers the ability to study how species have evolved in response to historical environmental changes, population bottlenecks, or shifting ecosystems (Hahn et al. 2020). This can deepen our understanding of genetic diversity and adaptability, allowing us to observe the historical baseline from which modern populations have diverged.

The expansion of genomic analyses to archival formalin‐preserved museum specimens will revolutionise multiple fields (Hahn et al. 2020). The interdisciplinary nature of archival epigenomics fosters enhanced collaboration between fields such as evolutionary biology, conservation science, epidemiology, and environmental genomics. The integration of chromatin profiling with genomic, proteomic, and transcriptomic data offers a multi‐omics approach that bridges traditional barriers between disciplines, enabling a holistic understanding of organismal responses to environmental change. Such collaborations can expand the scope and depth of research, bringing together expertise from molecular biology, ecology, and bioinformatics.

Museum ‘‐omics’ will provide evolutionary biology with unprecedented insights into historical genetic diversity and adaptation mechanisms. Epigenomic data enhance our ability to link genotype with phenotype, enabling studies on trait variation and adaptive mechanisms that offer a deeper understanding of species‐specific responses to change. Using museum specimens as a new source of temporal epigenomic data opens avenues for understanding how environmental stressors such as climate change have impacted gene regulation and organismal responses across different species. Accessing these time‐stamped epigenomic profiles across taxa allows for comparative analyses that can illuminate patterns of regulatory evolution and species‐specific adaptive mechanisms that operate on timescales too rapid for genome evolution alone to address. This is especially valuable for organisms with longer generation times, where epigenomic data can reveal their vulnerability or resilience to environmental shifts.

Conservation biology will benefit from understanding past population dynamics and responses to environmental changes, aiding current conservation efforts. The predictive potential of formalin‐preserved specimens cannot be overstated. By examining genome‐wide responses to past environmental changes, researchers can move beyond surface‐skimming summaries of epigenetic variation in contemporary wild populations and develop models that predict how species might respond to future environmental impacts, including climate change, habitat destruction, or the introduction of invasive species (Miryeganeh and Armitage 2024; Lamka et al. 2022).

Using museum specimens to gather historical gene expression data will not be without its challenges. Firstly, unlike genomic data, the epigenome is inherently specific to cell type and will vary significantly across tissues sampled from the same organism (Gibney and Nolan 2010). Thus, as is standard practice in RNA‐seq studies, great care must be taken to match the scientific question to the tissue most relevant to the trait of interest (Marco‐Puche et al. 2019). It will also be essential to consider sample size and design experiments with sufficient power for gene–environment associations to raise to the level of genome‐wide significance (Tsai and Bell 2015). Low statistical power in genome‐wide association studies (GWAS) has been a consistent issue in the field for decades (Li et al. 2014) and may be exacerbated further in epigenome‐wide association studies (EWAS) due to the expectation of greater noise in inherently environmentally sensitive genomic data (Flanagan 2015; Graw et al. 2019). Additionally, because chromatin accessibility is estimated via a read‐mapping approach, intra‐species genomic diversity that affects mapping efficiency could feasibly bias results, thus the choice of reference genome and the underlying genomic diversity in the study organism should be key considerations when interpreting results.

Because gene expression can vary rapidly in response to environmental stimuli, historical epigenetic studies will need to carefully consider the temporal and spatial context of collected specimens to avoid confounding factors such as seasonal or diurnal variation. To control for these effects, sample sets will need to be constructed to achieve consistency in metadata factors such as sampling date and collection location. Furthermore, developmental stage and tissue‐specific expression differences could introduce variability, requiring standardised sampling and analysis across specimens. Smaller sample sizes or uneven representation of certain taxa in collections may also limit the statistical power of some analyses. A final challenge to the broadscale adoption of historical epigenomics is the financial and computational burden of the approach. Estimating chromatin accessibility requires a higher sequencing investment than contemporary genomic approaches such as variant calling from whole‐genome skims. There are both technical and biological reasons for this. Initial data suggests that even in well‐preserved formalin specimens, only a proportion of the sequenced reads are expected to map to the reference (typically 20%–70%) (Hahn, Stiller, et al. 2024). Low mapping efficiency is due to low endogenous DNA content in samples (e.g. microbial biota will be co‐sequenced) and the inability of mapping algorithms to uniquely map short, low‐complexity reads, which make up a significant portion of historical datasets. Additional sequencing effort must be invested to compensate for these expected sources of data loss. Additionally, because the estimation of chromatin accessibility relies upon a peak‐detection approach (similar to ChIP‐seq), it is essential to sequence sufficiently deep to fully characterise the genome‐wide variation and reduce the possibility of false positives and negatives due to uneven sequencing within samples and unequal sequencing between samples. Thus, the resulting datasets are large (typically 150–500 million reads per sample) and have a concomitant requirement for access to high‐performance computing infrastructure for successful bioinformatic analysis.

With appropriate specimen selection and investment in sequencing depth, the molecular data from museums could be used to identify molecular sources of phenotypic variation as well as key genetic or regulatory markers associated with resilience or vulnerability, providing actionable insights for conservation management and policy (Balard et al. 2024). A temporal epigenomic approach may be especially impactful for species with low‐standing genetic diversity, such as endangered, recently introduced or clonal species, which likely rely on plastic and epigenetic responses to generate phenotypic diversity (Carneiro and Lyko 2020; Richards 2011). We might examine epigenetic biomarkers in comparison to historical profiles to estimate how reintroduced species are coping with modern environments or how introduced species adapt to novel environments and response to pest management actions (Gunn et al. 2024; Stuart et al. 2023). We might also examine epigenetic signatures in managed populations and use historical specimens to measure and minimise divergence between historically wild epigenotypes and those of captive‐bred individuals (Balard et al. 2024). Additionally, epigenetic insights could enhance provenance detection for harvested species lacking clear genetic population differentiation, providing local adaptation data that could inform sustainable practices in the fishing industry.

In human and wildlife medicine, studying historical pathogen‐host interactions can reveal patterns in disease emergence and spread. Preserved tissues and chromatin can simultaneously provide historical viral sequence data with which to conduct pathogen evolution analyses as well as insights into the host's immune response to past disease outbreaks or pathogen exposures, contributing valuable data to epidemiological models and helping to predict future disease dynamics (Speer et al. 2022). These insights can be especially valuable in the context of emerging infectious diseases or the re‐emergence of zoonotic pathogens, where understanding past responses could inform strategies for current wildlife and human health management.

Jointly, the fields of epigenetics and chromatin biology will be transformed by the ability to investigate historical chromatin states and gene regulation, providing a temporal dimension to our understanding of these processes. This integration of molecular techniques with museum genomics will unlock a treasure trove of historical biological data, vastly expanding our knowledge of genome diversity in underrepresented taxa and improving our mechanistic understanding of the plasticity of gene expression.

## Author Contributions

C.E.H. and E.E.H. conceived and wrote the manuscript.

## Conflicts of Interest

The authors declare no conflicts of interest.

## Data Availability

Data sharing is not applicable to this article as no new data were created or analysed in this study.
